# Teaching and assessing intra-operative consultations in competency-based medical education: development of a workplace-based assessment instrument

**DOI:** 10.1007/s00428-021-03113-6

**Published:** 2021-05-08

**Authors:** Marcio M. Gomes, David Driman, Yoon Soo Park, Timothy J. Wood, Rachel Yudkowsky, Nancy L. Dudek

**Affiliations:** 1 Department of Pathology and Laboratory Medicine, University of Ottawa, Ottawa, Canada; 2 Royal College of Physicians and Surgeons of Canada, Ottawa, Canada; 3 The Ottawa Hospital, Ottawa, Canada; 4 Department of Pathology and Laboratory Medicine, Western University, London, Canada; 5 Department of Medical Education, University of Illinois At Chicago, Chicago, IL USA; 6 Department of Innovation in Medical Education, University of Ottawa, Ottawa, Canada; 7 Department of Medicine, University of Ottawa, Ottawa, Canada

**Keywords:** Assessment, Workplace-based assessment, Validity, Intra-operative consultations, Entrustable professional activity, Competency-based medical education

## Abstract

Competency-based medical education (CBME) is being implemented worldwide. In CMBE, residency training is designed around competencies required for unsupervised practice and use entrustable professional activities (EPAs) as workplace “units of assessment”. Well-designed workplace-based assessment (WBA) tools are required to document competence of trainees in authentic clinical environments. In this study, we developed a WBA instrument to assess residents’ performance of intra-operative pathology consultations and conducted a validity investigation. The entrustment-aligned pathology assessment instrument for intra-operative consultations (EPA-IC) was developed through a national iterative consultation and used clinical supervisors to assess residents’ performance at an anatomical pathology program. Psychometric analyses and focus groups were conducted to explore the sources of evidence using modern validity theory: content, response process, internal structure, relations to other variables, and consequences of assessment. The content was considered appropriate, the assessment was feasible and acceptable by residents and supervisors, and it had a positive educational impact by improving performance of intra-operative consultations and feedback to learners. The results had low reliability, which seemed to be related to assessment biases, and supervisors were reluctant to fully entrust trainees due to cultural issues. With CBME implementation, new workplace-based assessment tools are needed in pathology. In this study, we showcased the development of the first instrument for assessing resident’s performance of a prototypical entrustable professional activity in pathology using modern education principles and validity theory.

**Supplementary Information:**

The online version contains supplementary material available at 10.1007/s00428-021-03113-6.

## Introduction

Competency-based medical education (CBME) has prompted a paradigmatic shift in medical education, with implementation mandated in multiple jurisdictions, including by the Royal College of Physicians and Surgeons of Canada (RCPSC) in Canada, the Accreditation Council for Graduate Medical Education (ACGME) in the USA, and the General Medical Council in the United Kingdom [1–5].

CBME differs from traditional models of learning, where a fixed time period is designated for training; in CMBE, residency training is designed around targeted competencies typically towards readiness for unsupervised practice and includes entrustable professional activities (EPAs) as units of work and assessment [6–9]. Assessment of competencies is considered a cornerstone for CBME to achieve its promise of better and safer health care outcomes [10–14]. Therefore, well-designed workplace-based assessment (WBA) tools will be required to document the competence of trainees in authentic clinical environments [15, 16].

Assessment in pathology is typically performed using remote end-of-rotation evaluations, which are not direct observations of a specific performance but rather reflect longer term observations of multiple facets of learning. Therefore, they do not directly reflect the ability to perform the required EPAs, which is a CBME requirement. Furthermore, residents’ performance is usually rated as a relative standard, either compared to their year of training or to their peers’ performance, but not using the CBME standard of readiness for independent practice.

The design of a new assessment tool aligned with CBME principles requires the incorporation of best practice in assessing real-life performance. There are a number of WBA instruments available for assessing specific clinical tasks using a variety of rating scales, including the Mini-CEX and the Objective Structured Assessment of Technical Skills (OSATS), among others [17–21]. Validity studies have shown that these tools perform better when they use construct-aligned rating scales [22–24]. With the operationalization of post-graduate training through EPAs [6–9, 25], Crossley argued that the construct that is being assessed is “entrustability” and demonstrated that entrustment-aligned scales increase reliability and generalizability of the educational measurement of clinical encounters [23, 24]. Similar results were noted in the assessment of procedural skills in the operating room [26] and bronchoscopy [27], and Rekman et al. proposed that entrustability scales should be used for competency-based clinical assessment [28].

The goal of this study was to develop a workplace-based assessment instrument to assess trainees’ performance of intra-operative pathology consultations, a prototypical anatomical pathology EPA with progressive entrustment of trainees.

## Material and **methods**

**Fig. 1:**
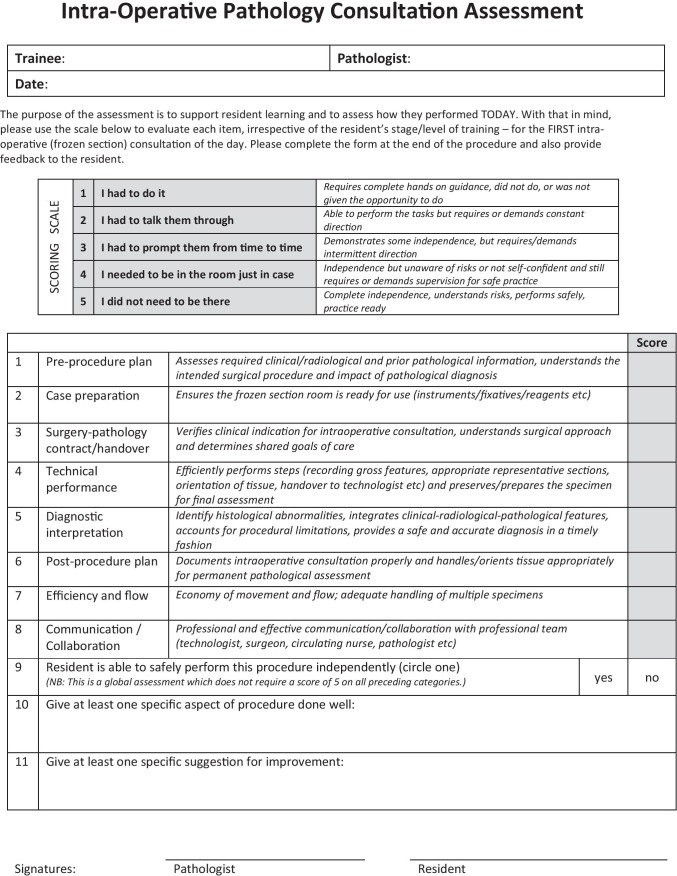
Entrustment-aligned pathology assessment instrument for intra-operative consultations (EPA-IC)

The entrustment-aligned pathology assessment instrument for intra-operative consultations (EPA-IC) is developed in 2015 and introduced at Western University’s Anatomical Pathology training program in 2016 (Fig. 1). It was used by clinical supervisors as part of the regular formative WBA of PGY-2 to PGY-5 residents’ performance of intra-operative consultations (PGY-1 s do not participate on intra-operative consultations at this residency program). Data was collected between May 30, 2016, and June 06, 2017.

### Sources of validity evidence

We used modern unified validity theory as a framework to guide the assessment development process and gather validity evidence [29, 30]. Validity is defined as the extent to which an assessment accurately measures what it is intended to measure. We assessed validity using the following sources of evidence, as proposed by Messick: (1) content, how the items on a tool were developed; (2) response process, whether raters and learners understand the task and are using the tool as expected; (3) internal structure, the psychometric properties of the assessment tool; (4) relations to other variables, the degree that the results of an assessment tool are related to other variables in expected ways; and 5) consequences, the impact the assessment tool will have, particularly on learners [29, 31, 32].

#### Content (design of the EPA-IC)

An experienced pathologist with special interest in medical education (MG) reviewed the literature related to best practices of intra-operative consultations [33, 34] and reviewed the O-SCORE tool [26] to identify the essential components required in a tool to assess resident’s performance. It is succinct, and the rating anchors are linked to readiness for independent performance of the procedure rather than performance relative to year of training.

The instrument was iteratively refined through: (1) consultation with an assessment expert; (2) University of Ottawa pathologists’ and residents’ feedback; (3) feedback from residents and pathologists who attended a national workshop and rated trainee's performance on video recorded simulated scenarios (approximately 60 participants); (4) Canadian pathology experts’ and residents’ survey feedback on the revised instrument; and (5) Consensus agreement by the authors (MG, DD, ND).

The EPA-IC (Fig. 1) is as an 11-item-instrument that assesses residents’ competence performing intra-operative consultations from the case preparation to the post-procedure plan. In addition to diagnostic interpretation and technical performance, attention was given to patient safety aspects, including tissue handover, communication, and collaboration skills. It included 8 items rated on a 5-point scale, one yes/no question regarding the trainee’s readiness to practice independently, and two open-ended questions asking about one specific aspect of the case performed well and one requiring improvement. The rating anchors were based on the rater’s judgment of trainee’s required supervision and support level, and ranged from 1 = “I had to do” (i.e., trainee required complete hands-on guidance or did not do the procedure) to 5 = “I did not need to be there” (i.e., trainee had complete independence and is practice-ready).

The focus groups also explored participants’ experiences with the EPA-IC, its content, and the specific items that were assessed.

#### Response process

During the academic year of 2016–2017, residents covering intra-operative consultations had their performance assessed by clinical supervisors using the EPA-IC. The new assessment instrument was presented to supervisors and residents in a 90-min workshop. There was no rater training because raters were reporting on their own behavior. Assessment was planned to take place immediately after the first intra-operative consultation of the day, with immediate feedback by the supervisor. EPA-IC forms were sent to the program coordinator for documentation. The program coordinator anonymized the forms, and the research assistant entered the data in a spreadsheet. Descriptive statistics were conducted to provide information about individual items’ performance, and focus groups with residents and raters were conducted to explore format familiarity, sources of biases, potential solutions to poorly performing items and biases, and the consequences of assessment.

(Appendices 1and 2 ). The focus group discussions were audio recorded, and the anonymized transcriptions were coded by two authors (MG and DD). Final codes were decided by consensus, described in a codebook, and iteratively applied to the transcripts. Emergent themes were recorded and iteratively interpreted by the authors.

#### Internal structure

Descriptive statistics, inter-item, and item-total correlations were analyzed. A generalizability study was performed to assess the reliability of the educational measurements. This model also determines how different variables contributed to the variability of the ratings, with the variance attributed to each variable expressed as a percentage of the overall variability in the ratings. Variance components were estimated using urGENOVA (Iowa City, IA). Statistical analysis was performed using SPSS.

#### Relations to other variables

Resident’s performance was compared to their year of training, which provides known-group validity evidence as relations to other variables. We determined the average rating across the scaled-response items to create a total procedure score for each trainee per procedure. We used total procedure scores in a series of factorial ANOVAs to study the effect of PGY level and whether residents were deemed ready to perform the procedure independently.

#### Consequences

Aspects related to the acceptability of the assessment by residents and supervisors were explored in the focus groups. An inductive thematic analysis was conducted to understand the impact of the EPA-IC on workload, workflow, and resident’s performance.

## Results

A total of 90 assessments were completed by 23 supervisors while observing 13 residents performing intra-operative consultations over a period of 12 months. Some items had missing data so 17 incomplete observations were excluded to keep a balanced design for analysis, leaving 73 complete observations of 12 residents (PGY2 = 5, PGY3 = 1, PGY4 = 4, PGY5 = 2; average 6.08 forms per resident; standard deviation 4.43, range = 1–17).

Sixteen participants accepted the invitation to participate in the focus groups, and three groups were organized: two focus groups with supervisors (*n* = 10; 5 male) and one focus group with residents (*n* = 6; all male).

### Content

Residents and supervisors commented that the EPA-IC included important components of intra-operative consultations and served as a checklist for “best-practices” and assessment.

However, items 2 (case preparation) and 7 (efficiency and flow) were missing a substantial number of ratings, which raised the possibility that some of the content of the EPA-IC is not representative of residents’ performance of intra-operative consultations or cannot be assessed by the supervisors. Some supervisors commented that the tasks under “case preparation” are usually performed by a technologist, as a delegated medical act. However, residents perceived value in performing such tasks for their own learning and for increasing the safety of the procedure. Regarding “efficiency and flow”, the focus group data indicated that the main issues were related to the response process (see below).

### Response process

A number of potential sources of rater and selection bias were identified in the focus group data analysis. Rater biases are an important component of the response process because raters might not be responding to assessment prompts as expected. Table 1 provides a summary of different types of rater bias [35]. Selection biases might also inflate or deflate ratings depending on the underlying reasons.

**Table 1:** Types of rater bias^a^

Type of rater bias	Description
Halo effect	A single score in a rating scale is awarded, which is designed to reflect the overall quality of the performance
Extreme response bias	The respondents may mark the extreme anchors rather than those in between, which can be due to other biases (see below)
Leniency-stringency bias	Some raters tend to be more lenient, while others are more stringent, which is usually related to personality traits
Incompetence bias	The rater tendency to assign high ratings because of his/her lack of confidence or competence in rating the behavior. This occurs when raters are incompetent on the tasks being rated, because they do not want to penalize the person being rated for his or her own shortcomings
Buddy bias	The degree of acquaintance between supervisor and trainee might increase ratings because of social aspects
Back-scratching bias	A faculty member gives high ratings to residents on the assumption that the resident will be less likely to give them a low rating (fear of retribution)

^a^Adapted from Berck RA^35^

There was a focus on “diagnostic interpretation” to the potential detriment of other aspects. This was associated with some biases, particularly the halo effect, in which different items were given less importance and scored equally together:

I was saying because some of those are a package together, all of them except the diagnostic, they actually work together. So if you are efficient with good turnaround times, you know what you're doing and how you handle the specimen, right? … if you’re bad in one, you're going to be bad in everything, right? I think so. Except the diagnostic [interpretation], which has multiple parts in it. - Supervisor

“Case preparation” and “efficiency and flow” were missing a number of ratings, and a number of biases identified by supervisors and residents were directly related to these items. These biases were usually related to the inability of the supervisor to assess these items, which resulted in overrating as a way not to be unfair to the learner (so-called incompetence bias):

To be honest, it’s because often they[supervisors] don’t check either. I think realistically if they’re not going to check their agents and they don’t see it as an important thing, they’re not going to ask the residents if they’ve done it right … - Resident

Interestingly, “efficiency and flow” was perceived by some supervisors as a personal trait, not as an ability that can be assessed and developed by the learner through training and coaching:

And then for the efficiency and flow, that could be a bit personal because it might be something to do with a relative ability or disability for an individual. And if they were a little bit slow for a variety of reasons or just inefficient for a variety of reasons, maybe that just seem a bit personal to be sort of remarking, ‘Boy, you were kind of slow’. - Supervisor

Leniency and buddy biases were overtly admitted by supervisors and perceived by residents. These biases frequently overlap and, for some supervisors, seem to be embedded in the culture of pathology. Supervisors sometimes did not fill out the EPA-IC when the resident had a poor performance on the first intra-operative consultation of the day. Others raised the possibility that residents might be self-selecting their better performances or performing differently when they know they are being assessed (so-called staged performance). These selection biases added to the inadvertently introduced selection bias of assessing residents’ performance on the first intra-operative consultation of the day, which also seem to have inflated the ratings:

Actually the first is often not a difficult one. It’s usually a margin or something. Sometimes more difficult ones come later in the day. - Supervisor

Additionally, some supervisors were not familiar with the format of the instrument and the rating scale, and stated that they were assessing residents in relation to their year of training (norm-referencing) rather than in relation to the entrustment that actually happened (criterion-referencing). Other supervisors and residents described the rating scale as more accurate, behavior-based, and less judgmental.

We also investigated whether the tasks being performed were too easy, even for junior learners. Supervisors unanimously agreed that residents are not ready for performing intra-operative consultations independently before PGY-4 or PGY-5 and once again reinforced that “diagnostic interpretation” is the skill that is ultimately being assessed.

### Internal structure

Mean item ratings (item difficulty) ranged from 4.41 to 4.89, but most of the items had some “1” and “2” scores assigned. The item-total correlations (item discrimination) ranged from 0.69 to 0.78, suggesting that items were able to differentiate between high- and low-performing trainees, but some of the items are producing similar ratings (Table 2). The analysis of inter-item correlations showed that “surgery-pathology contract/handover” and “efficiency and flow” were highly correlated (0.83).

**Table 2:** Descriptive statistics for the entrustment-based pathology assessment of intraoperative consultations

	Rating		Range		Item-total
Item	Mean	SD	Min	Max	Correlation
Pre-procedure plan	4.78	0.58	2	5	0.71
Case preparation	4.75	0.80	1	5	0.72
Surgery-pathology handover	4.77	0.68	1	5	0.78
Technical performance	4.58	0.88	1	5	0.72
Diagnostic interpretation	4.41	0.98	1	5	0.77
Post-procedure plan	4.71	0.63	2	5	0.78
Efficiency and flow	4.84	0.50	2	5	0.77
Communication/collaboration	4.89	0.36	3	5	0.69

A total score was generated by taking the average of the 8 items. The mean score and standard deviation of the evaluations was 4.72 ± 0.55. For the yes/no item that asked about the trainee’s readiness to safely perform the procedure independently, the distribution of scores was roughly equal: 56 (77%) of the 73 procedures or observations were marked as “yes”, and 17 (23%) were marked as “no”.

Table 3 displays the variance components of the different factors. Residents accounted for 5% of total variance. Forms within resident accounted for the most variance (48%), which indicates that there was variability within any resident as a function of the cases that they handled. Similar to the items analysis above, factors involving items accounted for low variability in the scores, indicating that the ratings of different items were similar, overall, and within any resident. The reliability of the performance assessment (G-coefficient) using this rating scale with an average of 6.08 observations/resident was 0.41. It is also possible to derive a generalizability coefficient that corresponds to the internal consistency of the scale. The resulting coefficient is 0.91 and supports the observation that the item ratings are similar.

**Table 3:** Results of G-study: variance components of the different factors

Facet	Variance	%Variance	Variance associated differences
p^a^	.032	5	Between residents
f:p	.281	48	Between forms any given resident received
i	.026	5	Between items
pi	.003	0	Residents getting different ratings on the items
fi:p	.243	42	Due to the interaction of all 3 factors plus overall error

^a^*p* resident, *f* forms, *i* items

G (overall) = (var(p) + var(pi)/ni)/(var(p) + var(pi)/ni) + var(f:p)/nf + var(fi:p)/nfni = .41

G (internal consistency) = var(p) + var(f:p)/(var(p) + var(f:p) + var(pi)/ni + var(fi:p)/ni = .91

### Relations to other variables

The mean score by year of training are summarized in Table 4. A between-subject ANOVA with PGY level as a between-subject factor showed a significant effect of PGY year [*F*(3,69) = 5.627, *p* = 0.002, partial eta square = 0.20]. The post-hoc *t* test (bonferroni) showed that ratings for PGY2 were lower than all others, PGY3 (*p* = 0.008) and PGY4 (*p* = 0.04). There was no significant difference between scores for PGY-3, 4, and 5. However, there was only one PGY3 in the cohort, which might have skewed the data if the PGY3 was a high performer among PGY3s.

**Table 4:** Overall performance according to PGME year of training

PGY^a^	Mean	SD	*N*
2	4.46	0.70	35
3	4.96	0.09	17
4	4.99	0.04	9
5	4.90	0.27	12
Total	4.71	0.55	73

^a^ Post-graduate year of training

The last question asked a global yes/no rating if the trainees could perform independently. The correlation between mean scores and whether the trainee could perform independently showed moderately high association, *r* = 0.62, *p* < 0.001. Table 5 shows the frequency of “yes” and “no” responses by PGY level. The overall pattern was that increases in PGY level leads to more “yes” responses on this item. Interestingly, the PGY2s and the PGY3 were not rated as “ready for independent practice” even when their ratings were “5” or close to it, in agreement with the supervisors’ “gestalt” that residents are not ready before PGY4-5.

**Table 5:** Ratings of resident ability to safely perform intraoperative consultations independently according to post-graduate year of training

Post-graduate year					
	2	3	4	5	Total
No	16	1	0	0	17
Yes	19	16	9	12	56
Total	35	17	9	12	73

### Consequences

Residents and supervisors accepted and welcomed the implementation of the EPA-IC. Two themes related to consequences emerged from our inductive thematic analysis.

#### Outcomes of assessment

##### Practice:

Residents and supervisors did not perceive any significant impact on workload. A couple of supervisors thought that there was some impact on the workflow and/or an increased cognitive load while performing intra-operative consultations but highlighted that the benefits were worth the effort. Many residents commented on the positive impact that the implementation of the EPA-IC had on their learning and practice, including becoming more deliberate in following a stepwise approach to intra-operative consultations:

I know I became much more systematic about the frozen sections because we’re being evaluated on different components of it so it’s not only just to screen the OR list the day before, but when you go in, you look at the room, you do all your checks for quality and for pre-analytics to make sure the room’s prepared, everything’s set. It really kind of pushed residents to play a much more active role in the procedure… - Resident

While many did not see any impact on the overall performance of intra-operative consultations, some residents and supervisors perceived an increase in the safety of the procedure as a consequence of the use of the EPA-IC as a checklist.

##### Instruction:

The participants were unanimous in saying that there were changes to the coaching process in the workplace. Residents noticed increased observation of their performance and increased quantity and quality of feedback by supervisors. Interestingly, some supervisors said that the changes were mainly to the observation, while others perceived more changes to the feedback:

And I think it helps assess other parts of the process that normally we gloss over. Like, at least one thinks it’s a given that they should have looked up the history and everything, and one focuses more on the interpretation of the actual gross or frozen section slide. And this kind of incorporates all the steps and itemizes things. And so you kind of get a better perception of the different steps of the process. - Supervisor

And.

But I do find that, although maybe you’re not observing things differently, you’re delivering feedback to them a lot differently. Because they’re getting it broken down what they did well and what they can improve on. – Supervisor

In general, the narrative comments written for items 10 and 11 of the EPA-IC were of poor quality. The majority of comments was not specific or behavior-based, did not validate or qualify positive aspects, and did not contain actionable feedback. In the focus groups, some supervisors commented on their inability to write narrative comments, while others did not want to document poor performance or improvement suggestions that could be perceived as criticism.

#### Entrustment of trainees

Residents and supervisors did not notice significant changes to the entrustment of trainees after implementation of the assessment. They commented on different aspects of entrustment, including factors related to the context, task, supervisor, and resident, but there was no comment about the relationship between the supervisor and the resident. For instance, there were many comments on how entrustment varied according to the difficulty and complexity of intra-operative consultations, as well as how entrustment varied according to resident seniority.

The entrustment process seems to be deeply embedded in the culture of pathology and the identity of pathologists. Although residents are fully entrusted to perform some tasks of intra-operative consultations independently, diagnostic interpretation and the communication of the diagnosis to the surgeon are perceived as more challenging, and there is open reluctance to ever fully entrust a trainee to make a diagnosis on their own:

So, we usually let the resident call the OR when it’s like straight forward. But when it becomes kind of tricky, you need some real communication, it would be the pathologist who will call. Usually when it’s like a grey zone, I don’t know what’s that, the situation needs real communication skills, usually we don’t let the resident call the OR. - Supervisor

Sometimes, this reluctance has roots in the relational identity of pathologists, as in expectations of the surgeons towards pathologists. Interestingly, it is perceived by supervisors that this reluctance to fully entrust trainees while in training could have an important negative impact on trainees and society:

And, you know, we have two PGY5s now who passed their exams and they’re still not going out on their own, right? They have a pathologist there to backup but we still never send them, right? Next week they could start practicing in the community and calling the frozens but we don’t. And I think this tool could help, once they met the competencies and they’ve written their exam. We should be doing that before we phase them out to the world. - Supervisor

## Discussion

With the implementation of CBME in multiple jurisdictions and specialties, well-designed workplace-based assessment instruments are needed to obtain a valid assessment of trainees’ performance on different EPAs. This study describes the development and the supporting validity evidence for assessing the performance of anatomical pathology trainees in the workplace while performing intra-operative consultations, a prototypical pathology EPA, using modern validity theory.

### Content

The construct being assessed in this study is the resident’s performance of intra-operative consultations. The intra-operative consultation literature largely focuses on diagnostic accuracy and microscopic interpretation, and best-practices studies are restricted to expert opinion, which were considered in the EPA-IC design [33, 34]. Intra-operative consultations are one of the RCPSC EPAs, one of the ACGME patient care sub-competencies and one of the EPAs proposed by the College of American Pathologists Graduate Medical Education Committee, and the EPA-IC items reflect many competencies included in these frameworks [8, 36, 37]. The design of our instrument incorporated the feedback of pathology residents and supervisors and assessment experts. The pilot study revealed one potential irrelevant item (case preparation) that is not considered a pathologist’s task by supervisors.

### Response process

A number of rater and selection biases were identified in our study, in large part due to a “lenient culture”. Also, some supervisors had the tendency to use the rating scale to judge performance against the level of training – as a norm-referenced Likert scale – instead of judging performance against the absolute standard of the entrustment decision that actually took place.

Physicians have historically put excessive emphasis on medical knowledge and expertise, which was in part responsible for unsafe practices that led to the development of the ACGME and RCPSC competency frameworks. In that sense, pathologists have focused on diagnostic interpretation and paid less attention to other tasks that are essential to perform safe intra-operative consultations (so-called soft skills, or intrinsic roles). Pathologists might attribute high ratings to these “soft skills” because they are not aware of them and do not feel confident or competent to rate them (so-called incompetence bias). Interestingly, diagnostic interpretation was the item with the lowest score and highest standard deviation, indicating that pathologists were more willing to give lower marks. This “diagnostic supremacy” along with the other rating issues indicates that raters and learners did not understand the task well and were not using the tool as expected or responding accurately to the assessment prompts.

We did not conduct rater training as previous studies using entrustment-aligned rating scales suggested that they are intuitive enough for expert practitioners to use. The criterion-based standard used is the ability to perform the tasks independently and, in theory, experienced practitioners should be able to judge it. However, it seems that supervisors were not aware of many of the tasks that they needed to observe and evaluate. In other words, the standard was not set as initially hypothesized, and rater training would have been helpful.

The issues discussed above indicate construct-irrelevant variance, or systematic error that is not related to the actual construct that is being assessed, which is one of the main sources of validity threats (Table 6) [38].

**Table 6:** Threats to validity in assessment

Construct-irrelevant variance	The variation in scores is due to something unrelated to the construct intended to be measured. For instance, if raters are considering the resident’s year of training when judging their performance, it could alter the score in a way unrelated to their ability to perform intra-operative consultations
Construct underrepresentation	Only part of the construct intended to be measured is actually being measured. For instance, if the ability to communicate results to surgeons is not assessed, the score would not capture all the aspects related to the ability to perform intraoperative consultations

### Internal structure

Our results show that the residents’ ratings were similar and quite high, even for junior trainees. These results are surprising, given that intra-operative consultations are regarded as a complex and stressful diagnostic task of anatomical pathologists. The restricted range in performance between residents is the main reason for the low reliability of the educational measurements. There are a number of possible explanations. The number of evaluations per resident and the number of residents per group is low, which contributes to undersampling [38]. Based on supervisors’ opinion, it does not seem that the tasks that are being evaluated are so basic that even residents at PGY2 level are capable of performing them well. However, it might be that the ability that actually discriminates resident’s performance is not being properly measured, which would correspond to construct underrepresentation. For instance, a majority of items could be easy to learn and not have a developmental trajectory, while others might be very complex, with the easy components lifting up the global ratings. Maturation did not seem to play a role, with PGY2s getting high scores since the beginning of the academic year (and they are not exposed to intra-operative consultations during PGY1). This lack of discrimination is more likely explained by a combination of rater and selection biases, and lack of rater training, as discussed above.

The different items are highly correlated with each other, which indicate that they are measuring the same construct from a psychometric standpoint. High inter-item correlation could be secondary to the high ratings observed for all items and potentially a consequence of the different biases previously discussed. Alternatively, it could be that items are worded in a way that they are capturing similar information or they are not capturing the discriminating aspects of trainee’s performance on the different tasks. Given the fact that completely distinct tasks that require different skill sets were rated the same way, the latter explanation is less likely.

The high item correlations also suggest that the scale could be reduced to one item from a psychometric standpoint, although, in doing so, the opportunity to provide specific feedback would be lost.

The lack of reliability is a threat to validity. Even though the main purpose of WBA is formative, the inability to discriminate good and bad performance might prevent the diagnosis of learners’ needs, limit the opportunities for coaching feedback, and fail to document the developmental growth of learner’s competence. Therefore, this issue needs to be addressed in future studies.

### Relations to other variables

PGY2s had lower ratings than PGY3-5 residents and were less frequently considered ready for independent practice. However, the difference in ratings was of small magnitude.

Although the supervisors’ opinions suggest that residents only achieve readiness for independent practice by PGY4-5, the single PGY3 in the study had similar overall ratings to the seniors. Nevertheless, the PGY3 could happen to be a high performer which might have skewed the results and does not allow us to make any conclusion.

The fact that PGY2s and the PGY3 were not rated as “ready for independent practice” even when their ratings were high might be because a critical item (such as diagnostic interpretation) does not mature until later but also might suggest that faculty are actually basing their decision more heavily on trainee level rather than their observed performance.

### Consequences

The implementation of the EPA-IC had an important impact on residents’ learning. It increased direct observation and the amount of feedback, and made feedback more specific. The new assessment was well accepted by residents and supervisors, with a few of them reporting improvement in the practice of intra-operative consultations. Since the main purpose of WBA is to provide frequent, specific, and actionable feedback to learners so that they can progress in their developmental trajectory towards readiness for independent practice, these results remain a strong argument for the validity of the EPA-IC.

No significant changes were noted in the entrustment process, which seems to be limited by cultural norms. Diagnostic accuracy is an important part of the pathologist’s identity, and supervisors are reluctant to fully entrust a trainee to do it independently. However, these cultural norms, particularly those that relate to the communication with surgeons, need to be addressed because they might have a negative impact on patient safety as residents transition to independent practice.

### Limitations and next steps

This study has some limitations, including the low sample size, the low number of residents per group (post-graduate years), and the variation in the number of assessments per resident with many residents having a single assessment. All these aspects limit the interpretation of the psychometric analysis. Also, the study was done in a single residency program, and variations in contexts and practices could not be investigated. As suggested by our qualitative data, culture and identity play an important role in multiple aspects of assessment; therefore, results cannot be generalized to other countries or maybe even other residency programs in Canada.

Efforts are underway to address some of the threats to validity that were identified in our pilot study. The instrument and its items need to be revised according to our initial findings, the sample size needs to be increased, frame-of-reference rater training needs to be offered, and other institutions need to be involved.

## Conclusion

With CBME implementation, new WBA tools are needed for assessing pathology EPAs [5, 8, 9, 39]. We conducted a pilot study using a newly developed WBA instrument for assessing residents’ performance of intra-operative pathology consultations, a prototypical pathology EPA, and we presented the validity evidence that supports the use of the results of assessment. The content is appropriate, the assessment is feasible and acceptable to residents and supervisors, and it had a positive educational impact of making explicit the necessary steps to successfully perform the EPA, as well as increasing observation of and feedback to learners. The low reliability of the results is the main threat to validity and seems to be related to response process issues. Given the low stakes and formative nature of WBA, the educational impact on learners should be emphasized by faculty development activities that focus on coaching strategies, and valuing narrative comments over rates. Future studies will address the threats to validity identified. However, since some of the threats seem to be deeply embedded in the culture of medicine and pathology, one should not expect to see rapid changes and should approach WBA and CBME implementation through a quality improvement lens: with formative rather than summative purposes.

## Supplementary Information

Below is the link to the electronic supplementary material.

Supplementary file1 (DOCX 18 KB)

Supplementary file2 (DOCX 15 KB)

Supplementary file3 (XLSX 22 KB)

## Data Availability

The datasets used and/or analyzed during the current study are available from the corresponding author on reasonable request.
