# Supplementary material for: Teaching and assessing intra-operative consultations in competency-based medical education: development of a workplace-based assessment instrument
Source: Virchows Arch. 2021 May 8;479(4):803–13. doi: 10.1007/s00428-021-03113-6 (PMC8516791; doi:10.1007/s00428-021-03113-6)
Supplement: Supplementary file 2 — Supplementary file2 (DOCX 15 KB) [file 428_2021_3113_MOESM2_ESM.docx]

Protocol Title: Development of a Workplace Direct Observation Assessment Tool of Technical Skills in Pathology (ID: 7028)

(Note: all iterative qualitative research adapts and changes to best serve the study goals and the particular needs of the participants. Therefore, some changes to the Questionnaire are inevitable during the course of the Focus Group. This template serves as a guideline only).

Semi-Structured Focus Group Questionnaire (Supervisors)

*Thank you for agreeing to participate in our focus group today exploring your experiences with the Entrustment-aligned Pathology Assessment Tool (we will be calling it EPAT for convenience). First of all, please be sure that you have signed your consent form.*

*Please note that this group is being audio-recorded and will be transcribed professionally prior to analysis. Any names or identifying details that may be mentioned will be removed and only de-identified data will be shared with the study team. In order to respect your colleagues’ confidentiality, please refrain from discussing this Focus Group with non-participants. Do you have any questions before we start? If not, let’s begin:*

1. How was the assessment of resident's performance on intra-operative consultations done on your department before the implementation of the EPAT?
   Prompt: Can you describe a typical assessment before the EPAT?
2. As compared to the previous assessment system, what changed with the implementation of the EPAT?

Prompt: Did it affect your daily routine when performing intra-operative consultations? If so, can you give me an example?

1. Did this implementation affect the quality or quantity of direct observation that you do when you're supervising residents performing intra-operative consultations? How so?
2. Did this implementation affect the quality or quantity of feedback that you give to the residents?
   Prompt: Can you give me an example?
   Prompt: Was face-to-face feedback more or less common than previously?

Prompt: If you did not see much impact, can you describe why you think feedback remained the same?

1. What items of the assessment tools are harder to assess? Why?
   Prompt: Can you give me a specific example of a time when it felt challenging to address an item on the tool?
2. Specifically, I’d like to ask you about Items 1 and 7. Item 1 is “case preparation” and Item 7 is “efficiency and flow”. They were less frequently rated than the others: why do you think this might be?
   Prompt: What kinds of things generally contribute to “skipping” items on an assessment tool?
3. The mean rating for the different residents were quite high. Why do you think that happened?

Prompt: What kinds of things do you think might contribute to giving high marks to most residents?

Prompt: Most PGY4s were assessed as ready for independent practice. Do you agree that PGY4s are usually ready to perform intra-operative consultations independently? If not, why do you think they were rated that way?

1. There is not much difference in the rating of the different items for a given assessment. Why do you think that happened?

Prompt: What kinds of things do you think might contribute to giving a similar mark to the different items?

1. In your opinion, what could be the impact on someone's practice with the implementation of the EPAT?
   Prompt: Could you describe potential positive impacts? Negative?
2. Is there anything else you’d like to share about your experiences using this tool or any suggestion for improving the tool?

*Thank you very much for taking the time to talk with us today.*
